# Specificity, Privacy, and Degeneracy in the CD4 T Cell Receptor Repertoire Following Immunization

**DOI:** 10.3389/fimmu.2017.00430

**Published:** 2017-04-13

**Authors:** Yuxin Sun, Katharine Best, Mattia Cinelli, James M. Heather, Shlomit Reich-Zeliger, Eric Shifrut, Nir Friedman, John Shawe-Taylor, Benny Chain

**Affiliations:** ^1^Department of Computer Science, UCL, London, UK; ^2^CoMPLEX, UCL, London, UK; ^3^Division of Infection and Immunity, UCL, London, UK; ^4^Department of Immunology, Weizmann Institute, Rehovot, Israel

**Keywords:** T cell receptor, repertoire analysis, ovalbumin, machine learning, CDR3

## Abstract

T cells recognize antigen using a large and diverse set of antigen-specific receptors created by a complex process of imprecise somatic cell gene rearrangements. In response to antigen-/receptor-binding-specific T cells then divide to form memory and effector populations. We apply high-throughput sequencing to investigate the global changes in T cell receptor sequences following immunization with ovalbumin (OVA) and adjuvant, to understand how adaptive immunity achieves specificity. Each immunized mouse contained a predominantly private but related set of expanded CDR3β sequences. We used machine learning to identify common patterns which distinguished repertoires from mice immunized with adjuvant with and without OVA. The CDR3β sequences were deconstructed into sets of overlapping contiguous amino acid triplets. The frequencies of these motifs were used to train the linear programming boosting (LPBoost) algorithm LPBoost to classify between TCR repertoires. LPBoost could distinguish between the two classes of repertoire with accuracies above 80%, using a small subset of triplet sequences present at defined positions along the CDR3. The results suggest a model in which such motifs confer degenerate antigen specificity in the context of a highly diverse and largely private set of T cell receptors.

## Introduction

The T cell compartment recognizes antigen using a large and diverse set of antigen-specific receptors created in the thymus by a complex process of imprecise somatic cell gene rearrangements. The clonal theory of immunity (1) proposes that lymphocytes carrying receptors that specifically bind an antigen to which the immune system is exposed, for example, during infection or vaccination, respond by proliferating and differentiating. This population of expanded and differentiated cells then confers on the system the acquired ability to respond specifically to the antigen to which it had previously been exposed. The clonal theory therefore explains the immune system properties of specificity and memory. A prediction of this theory is that the frequency of lymphocytes that have been exposed to antigen (i.e., memory or effector cells) will be greater than the frequency of those that have not (i.e., naive). This prediction has been verified for T cells in a wide variety of models, using antigen-specific readouts such as cytokine responses, and major histocompatibility complex (MHC) multimer binding to identify expanded lymphocyte clones (2–4). The selective expansion of specific clones has also been inferred from global measurements such as V region usage (5) or spectratyping (a technique sometimes referred to as the immunoscope) (6). The sequences of TCRs with a known antigen specificity have been examined previously, especially in the context of CD8+ T cells and influenza. The sequences of epitope-specific sets of TCRs were often very diverse, and many TCRs were private (i.e., found predominantly only in repertoires from one individual) (7). However, public TCRs (shared between repertoires of many individuals) were sometimes observed in responses to certain epitopes (8).

The introduction of high-throughput sequencing (HTS) has opened up new approaches to examining the diversity of TCR sequences at a global level. Each individual animal or human has been shown to contain T cells carrying billions of different receptors (9, 10) and several computational bioinformatic approaches have been developed which aim to capture the diversity and structure of the overall TCR repertoire (11–13).

We have previously used short read parallel HTS to estimate T cell receptor β transcript frequencies and sharing (14, 15), and to explore the global changes in the CD4+ T cell receptor repertoire following immunization (16). The latter study focused on local features of protein sequence within the TCRβ CDR3 loop, which interacts directly with peptide antigen lying within the MHC groove. The TCRβ CDR3 encodes the largest amount of sequence diversity, coded for by the combination of the D regions, and the DJ and VD junctions. We therefore mapped the sets of TCR CDR3β sequences from each animal to a lower dimensional feature space indexed by short stretches of contiguous amino acids (typically triplets). A Support Vector Machine (17), a classical regularized machine learning algorithm, was then able to distinguish between TCR repertoires of unimmunized mice and mice immunized with an extract of *Mycobacterium tuberculosis* [complete Freund’s adjuvant (CFA)] within the lower dimensional transformed feature space.

Complete Freund’s adjuvant contains a complicated mixture of protein and non-protein antigens and causes more widespread perturbations of the repertoire than single protein antigens. However, purified protein antigens are poorly immunogenic except when given in the context of adjuvants, which are believed to provide a danger signal which stimulates innate immunity and hence drives effective antigen presentation (18, 19). We therefore wished to extend our investigation to analyze the response to a well-studied model antigen, ovalbumin (OVA), when delivered in the context of CFA. Specifically, we wished to test the hypothesis that the frequencies of short amino acid motifs within the TCR CDR3 reflected the antigen specificity of the response and could be used to distinguish between repertoires of mice immunized with OVA plus adjuvant and those immunized with adjuvant alone.

Instead of SVM, we used linear programming boosting (LPBoost), an algorithm which minimizes a 1-norm soft margin error function (20). Unlike SVM, which uses a non-zero weighted combination of all features for classification, LPBoost typically selects a small number of features with non-zero weights. This significantly reduces the computational cost, particularly when dealing with very large numbers of features (21, 22). More importantly, feature selection gave biological insight by indicating which amino acid motifs within the CDR3 were most important in contributing to any specificity observed. The results of the LPBoost algorithm, either alone or in combination with SVM, achieved significant classification accuracy using a small sub-sample of amino acid triplet motifs. The frequency of small sets of conserved amino acid strings, often found toward the ends of the CDR3 loops, therefore, contained the information necessary to distinguish repertories of different antigen specificity.

## Materials and Methods

### Sample Collection and Sequencing

Thirty-three C57BL/6 mice were immunized with CFA, with or without an additional protein/peptide antigen. The primary antigen used in this study was OVA, a protein commonly used as a model antigen in the immunological literature (Sigma, Poole, UK). The mice were immunized in two independent experiments. The first set contained nine mice immunized with OVA + CFA and nine mice with CFA alone. Three mice of each class were culled at days 5, 14, and 60. The second set contained six mice immunized with OVA + CFA and four mice with CFA alone. Three OVA and two CFA mice were culled at days 7 and 60. Repertoires from a set of five mice immunized with a peptide coding for a sequence of the heatshock protein HSP60, VLGGGCALLRCIPALDSLTPANED (p277) (23) were also analyzed. After immunization, mice were sacrificed and spleens collected at either early time points (samples from days 5, 7, and 14 were combined for this analysis), or a late time point (day 60). Mice taken down at 60 days were given a booster of incomplete Freund’s adjuvant with or without OVA at day 14. Mice were housed at the Weizmann Institute of Science under conditions approved by the Institutional Animal Care and Use Committee in compliance with national and international regulations.

The ultra high dimensionality of the data precludes conventional power calculations; the minimum group size of five mice per group is toward the lower limit typically used for the application of the machine learning algorithms used in the study.

CD4+ T cells were isolated from spleens and TCRβ chains from these cells were sequenced *via* the protocol described in Ref. (14). Briefly, total RNA was reverse transcribed with a primer specific to the TCRβ constant region, and resulting cDNA was amplified *via* PCR using a set of TCRVβ primers. Illumina adaptors were ligated to the product, including indexes to identify each sample, and the sequencing was performed on a Genome Analyzer II. The repertoires were sequenced in a total of four sequencing runs, one with six OVA and six CFA run on each run (days 5 and 14 together); one run with three OVA and three CFA together (days 60 experiment 1); and two runs with three OVA + CFA and two CFA alone on each run (days 7 and 60).

### Data Preprocessing

Raw sequence data were analyzed and error corrected using a short read modification of Decombinator as described in detail previously (16). The fastq files are available at http://www.ncbi.nlm.nih.gov/sra/?term=SRP075893.

### Sequence Distance/Similarity Measures

In order to compare two CDR3β amino acid sequences, two measures are used. The Levenshtein distance counts the number of edits (insertions, deletions, or substitutions) that are needed to transform one of the CDR3s into the other. The p-spectrum kernel is a similarity measure, counting the number of substrings of length *p* that are shared between two CDR3s (24).

### Sequence Features

Each CDR3β sequence was mapped to a numeric feature space using string features. The string feature is the number of times (term frequency) each *p*-length substring (typically triplets, *p* = 3, number of features = 20^3^ = 8,000) appears in a set of CDR3 sequences (i.e., a repertoire).

### Linear Programming Boosting

Linear programming boosting is described in detail in Ref. (20). The classification method generates a classifier which is a linear combination of features and can be viewed as an example of soft margin maximization algorithms like SVM, but minimizing a 1-norm. Since the optimization problem only involves linear terms in both constraints and objective (as compared to classic L2 minimization) it reduces to a linear programme (LP). In order to reduce the potentially very high computational cost of solving the LP with many features, LPBoost makes use of the column generation technique by iteratively optimizing the dual misclassification costs and generating weak learners as new LP columns.

The algorithm is described in brief below. Let *H* denote an *m* × *n* matrix, where each column is one weak learner (a vector of string features as defined above) and each row is a different repertoire. Let *y_i_* ∈ {−1, +1} denote the label of the *i*-th data sample (for example, OVA immunized or non-OVA immunized). The primal form of the LP formulation is given by

$$\begin{array}{l} \underset{\alpha ,\xi ,\rho }{\text{max}}\;\rho -D\sum_{i=1}^{m}\xi_{i}\\ \text{subject to }y_{i}H_{i}a+\xi_{i}\geq \rho ,i=1,\ldots ,m\\ \;\sum_{i=1}^{n}a_{i}=1,\xi_{i}\geq 0,i=1,\ldots ,m\\ \;a_{j}\geq 0,j=1,\ldots ,n \end{array}$$

where *m* is the number of training data points, *n* is the number of dimensions (or weak learners or features), *H* is the set of features associated with each data point, *D* is the penalty for misclassification (set by user), ρ is the classification margin (learnt during the algorithm), ξ are the set of slack variables for data points within margin (learnt during the algorithm), and *a* are coefficients defining classifying hyperplane (i.e., feature weights learnt by the algorithm). *D* (and the misclassification penalty for the SVM discussed below) were chosen by randomly dividing the data for each mouse into two disjoint sets sharing no sequences; selecting optimum parameters using leave-one-out validation on one set of data, and then applying these parameters to the leave-one-out train/test validation sequence as described. In this way, we use one sample to select the parameters before effecting an independent leave-one-out estimation of the accuracy of classifiers trained with these parameters.

The dual form is

$$\begin{array}{l} \underset{u,\beta }{\text{min}}\;\beta \\ \text{subject to }\sum_{i=1}^{m}u_{i}y_{i}Hij\leq \beta ,j=1,\ldots ,n\\ \;\sum_{i=1}^{m}u_{i}=1,0\leq u_{i}\leq D,i=1,\ldots ,m \end{array}$$

where β is the objective function to be minimized, *u* are the sample weights.

Linear programming boosting assigns non-negative weights to both features and samples, but typically produces a sparse feature vector where only a few features (i.e., substrings) have non-zero coefficients. The LPBoost algorithm therefore serves as a feature selection step as well as a classification. We therefore experimented with combining LPBoost with SVM, thus combining the advantages of sparse feature selection (with associated improved biological interpretability) with the non-linear classification advantages of SVM. The code for LPBoost can be found at https://github.com/YuxinSun/LPBoost-Using-String-and-Fisher-Features.

### Data Repository

The raw fastq sequence files can be found at the Short Read archive (http://www.ncbi.nlm.nih.gov/sra/), accession number SRP075893.

### Other Analysis

Multiple sequence alignments were viewed in Aliview (25) which uses MUSCLE (26) for executing the alignment. The phylogenetic trees of the alignments were created using FastTree (27) and displayed using the APE package in R (28). Heatmaps were created using the *heatmap.2* function in the *gplots* R package.

## Results

### The OVA Expanded Repertoire Is Private but Shares Some Sequence Similarity

We analyzed CDR3β sequences from a total of 33 mice (8–12 weeks old), immunized with CFA emulsified with either PBS only, OVA dissolved in PBS, or the p277 peptide dissolved in PBS. The immunized mice were classified as “early” (days 5, 7, and 14) or “late” (day 60). CDR3β sequences from eight unimmunized mice and two mice injected with PBS only were also available.

**Figure 1:**
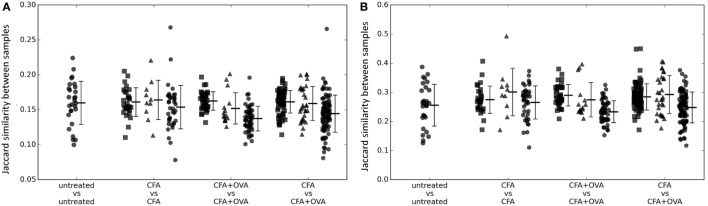
**(A)** The number of shared CDR3 sequences between pairs of mice of different immunization status (as shown on *x* axis) measured as the Jaccard index. Squares = early (days 5–14); triangles = late (day 60); and hexagons = early/late comparisons. **(B)** As for panel **(A)**, but calculated using only the top 5% CDR3s ranked according to frequency in each sample.

We first investigated whether exposure to OVA produced a detectable common CDR3 signature by measuring the overlap between the OVA immunized repertoires, and the non-OVA immunized repertoires (Figure 1A). However, there was no evidence that OVA immunized mice shared a greater proportion of CDR3s than were shared between any OVA immunized and any non-OVA immunized mouse. We hypothesized that the OVA-specific CDR3s might be enriched in the more expanded (i.e., higher frequency) set. However, the same result was obtained when sharing was evaluated using the most abundant 5% (Figure 1B) or 10% of CDR3s (Figure S1 in Supplementary Material) as when using the whole repertoire (Figure 1A).

**Figure 2:**
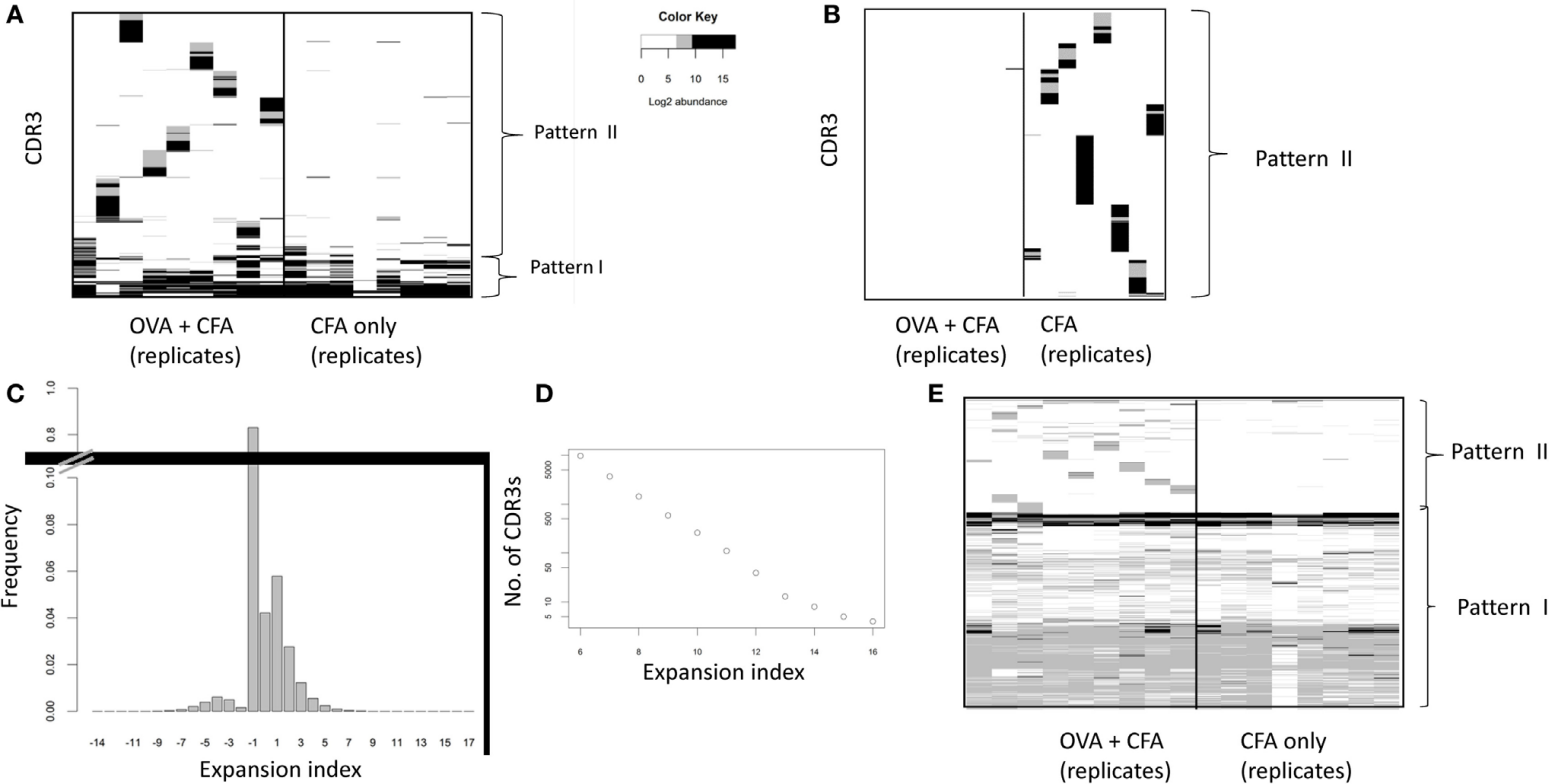
**(A,B)** The abundance of high frequency CDR3s across repertoires of ovalbumin (OVA) or complete Freund’s adjuvant (CFA) mice. The 100 most abundant CDR3s from each OVA + CFA “early” repertoire **(A)** or CFA only “early” repertoire **(B)** were selected, and their abundance (shown as Log_2_ counts/million) is shown in each OVA + CFA or CFA only immunized repertoire. Each column of the heat map represents one mouse repertoire. Each row of the heat map represents a distinct CDR3. Abundances below 2^6^ counts/million are in white. Pattern I indicates CDR3s which are abundant in both OVA + CFA and CFA only repertoires. Pattern II indicates CDR3s which are abundant in OVA + CFA repertoires. **(C)** The frequency distribution of the expansion index over all early immunized repertoires. The expansion index for each CDR3 is calculated as Log_2_ (abundance in a specific repertoire/average abundance in all unimmunized repertoires). For those sequences present in immunized repertories but absent from all unimmunized repertories, the abundance in unimmunized mice was set to 1 in 10^6^ for calculation of expansion index. // shows that the column height is truncated. **(D)** The number of CDR3s in all early immunized repertoires with an expansion index greater than the threshold shown. The expansion index for each CDR3 is calculated as Log_2_ (abundance in a specific repertoire/average abundance in all unimmunized repertoires). **(E)** The expansion index of all CDR3s with expansion index >6 in any OVA + CFA repertoire plotted for each OVA + CFA or CFA early immunized repertoires. Each column of the heat map represents one mouse repertoire. Each horizontal row of the heat map represents a distinct CDR3 with expansion index >6 in one or more OVA + CFA repertoires. Only those positions with an expansion index of >6 in that mouse are colored non-white. Pattern I indicates CDR3s which are abundant in both OVA + CFA and CFA only repertoires. Pattern II indicates CDR3s which are abundant in OVA + CFA repertoires.

In order to identify individual CDR3βs that were potentially shared by the OVA immunized repertoires we plotted the frequency of the 100 most abundant CDR3βs from each OVA + CFA mouse (Figure 2A) or the frequency of the 100 most abundant CDR3βs from each CFA only immunized mouse (Figure 2B). We focused our initial analysis on the “early” samples, reasoning that the clonal expansion was likely to be more pronounced during this period than 2 months after immunization. Two major patterns of CDR3 abundance could be observed in the OVA repertoires. The first rarer pattern consisted of CDR3s which were abundant in the repertoires of both OVA + CFA and CFA only immunized mice. These CDR3s are observed in more than one mouse and across both groups of mice. We hypothesize that these may represent abundant “public” CDR3βs which have been reported previously [e.g., Ref. (15)], and which do not reflect antigen-specific responses. The second pattern observed (represented by the blocs of sequences observed in each mouse) were CDR3βs that are highly abundant in mice that received the OVA + CFA immunization and are mostly absent in the group that received only CFA. These CDR3βs are private—they are found in one mouse and mostly absent from others. In CFA only repertoires, the 100 most abundant CDR3βs all showed pattern 1 (i.e., individual specific, and absent in OVA + CFA repertoire), perhaps reflecting that the stronger individual-specific responses to CFA in these mice reduce the rank of the public TCRs so they do not appear in the top 100 most abundant CDR3s.

In order to exclude the public CDR3s, and focus on potential antigen expanded CDR3s we calculated an “expansion index,” which measures the abundance of each CDR3 relative to its average abundance in unimmunized mice and is expressed in Log_2_ (Figure 2C). The number of sequences in CFA + OVA immunized mice with an expansion index above a given minimum threshold is shown in Figure 2D. We selected a threshold of six for further analysis, which is equivalent to a minimum of six doubling divisions assuming no death. This is a reasonable estimate for the maximum likely number of divisions in our earliest time point (5 days post vaccination) given available estimates of cell division times (29, 30). The expansion index of each of the 10,175 CDR3βs with an expansion index greater than 6 in any OVA + CFA repertoire, across all early immunized repertoires is shown in Figure 2E. The overall pattern observed remains the same as in Figure 2A, showing sets of individual expanded CDR3s that are mostly specific to each mouse (putative OVA-specific TCRs), plus CDR3βs common to all early immunized repertoires (putative public CFA-specific TCRs). The complementary plot of each of the CDR3βs with an expansion index greater than 6 in any CFA repertoire is shown in supplementary Figure S2 in Supplementary Material. The pattern is very similar, showing sets of individual expanded CDR3s that are mostly specific to each mouse (putative private CFA-specific TCRs), plus CDR3βs common to all early immunized repertoires (putative public CFA-specific TCRs).

**Figure 3:**
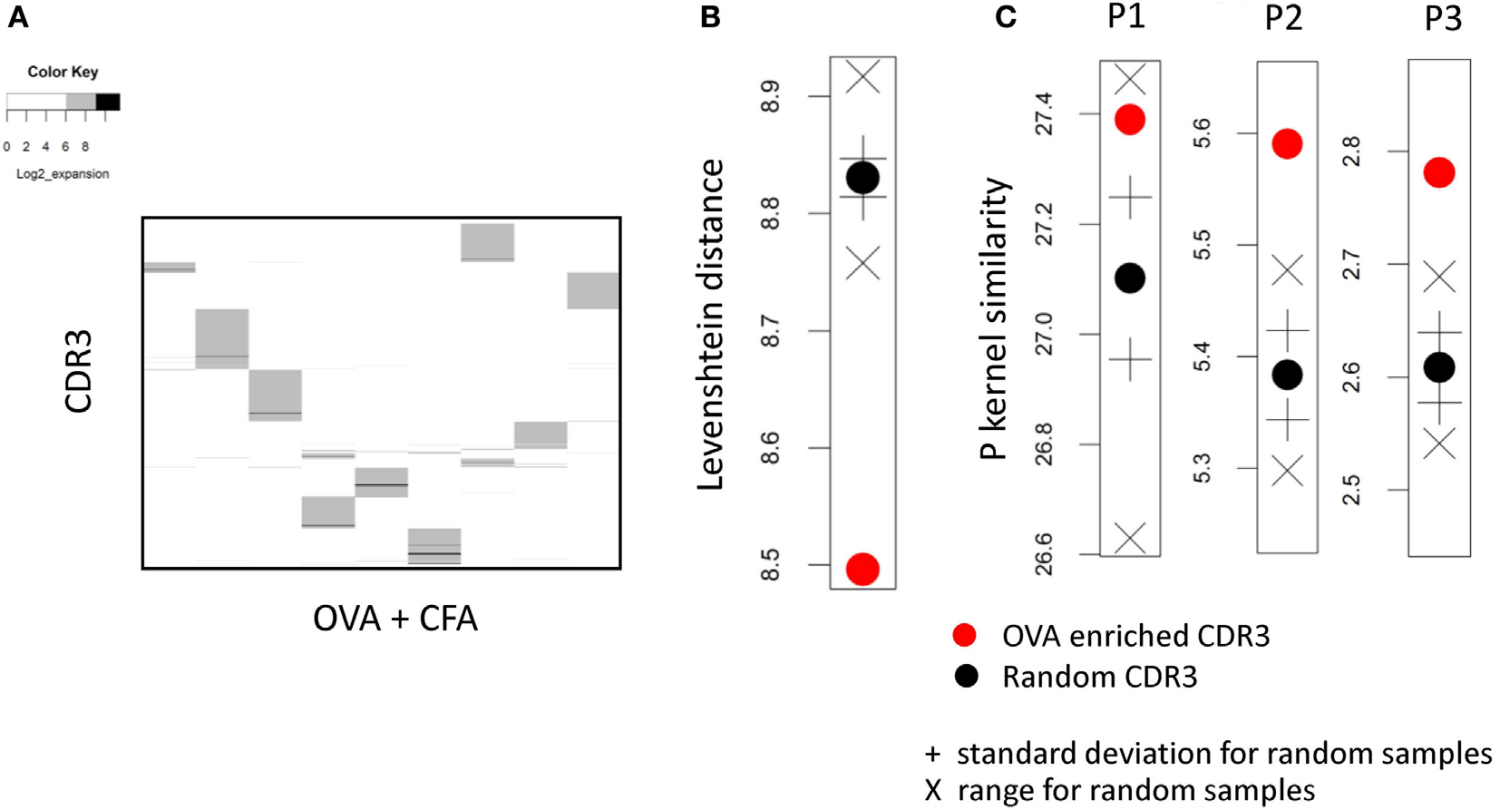
**(A)** The expansion index of all CDR3s with expansion index >6 in any ovalbumin (OVA) + complete Freund’s adjuvant (CFA) repertoire, but excluding CDR3s with an expansion index of >4 in any CFA repertoire. The expansion index is plotted for each OVA + CFA or CFA early immunized repertoires. Each column of the heat map represents one mouse repertoire. Each horizontal row of the heat map represents a distinct CDR3 with expansion index >6 in one or more OVA + CFA repertoires, but excluding CDR3s with an expansion index of >4 in any CFA repertoire. **(B)** The mean pairwise Levenshtein distance between OVA-associated (red circle) and 100 sets of random (black) CDR3s. + SD above/below the mean for random sets; X shows max/min for random sets. **(C)** As for panel **(B)** but showing the p-spectrum kernel pairwise similarity metric for *p* = 1, 2, 3.

In order to focus on the OVA-specific response, we further refined our CDR3 selection by excluding all CDR3βs with an expansion index >4 in any CFA only repertoire. Each CDR3β in this set therefore had an expansion index >6 in at least one OVA + CFA repertoire, and <4 in all CFA only mice. This left a total of 2,335 CDR3β sequences. The expansion index of these “OVA-associated” CDR3s across the repertoires of CFA and OVA + CFA mice is shown in Figure 3A. Importantly, we did not observe any public clones within this set; specifically there were no TCRs which satisfied these criteria (i.e., >6 in OVA but <4 in CFA) and were found in all (or even the majority of) OVA immunized mice. Instead the CDR3s with a high expansion index in OVA + CFA repertoires, but not in CFA repertoires are almost completely private.

We next investigated whether the set of OVA-associated CDR3s we identified above shared any detectable sequence similarities. We measured the sequence distance (the Levenshtein distance, defined as the number of additions, deletions, and substitutions required to go from one sequence to another) between all pairs of CDR3βs within this set, and compared these to 100 sets (of the same size) of CDR3βs selected randomly from the combined repertories of all mice (Figure 3B). The CDR3s from the OVA-associated set showed a small but consistent decrease in pairwise distance compared to random sets. We also measured similarity between the CDR3s using the *p*-length string kernel (a measure of sharing of *p*-length contiguous amino acid substrings between CDR3s). The results, again compared to 100 random CDR3 sets, for *p* = 1, 2, and 3 are shown in Figure 3C. The CDR3s from the OVA-associated set showed a small but consistent increase (note this is now a similarity metric) in pairwise similarity compared to random sets. This increased similarity was more pronounced for *p* = 2, 3 than *p* = 1 suggesting that short amino acid motifs rather than simply single amino acid abundance characterized the OVA-associated set of CDR3βs. Taken together these results suggest that the CDR3s with a high expansion index in OVA + CFA repertoires and low expansion index in “CFA only” repertoires share some sequence features.

### Linear Boosting Can Distinguish between CFA + OVA and CFA Only Repertoires, Using a Small Number of Amino Acid Motifs

**Figure 4:**
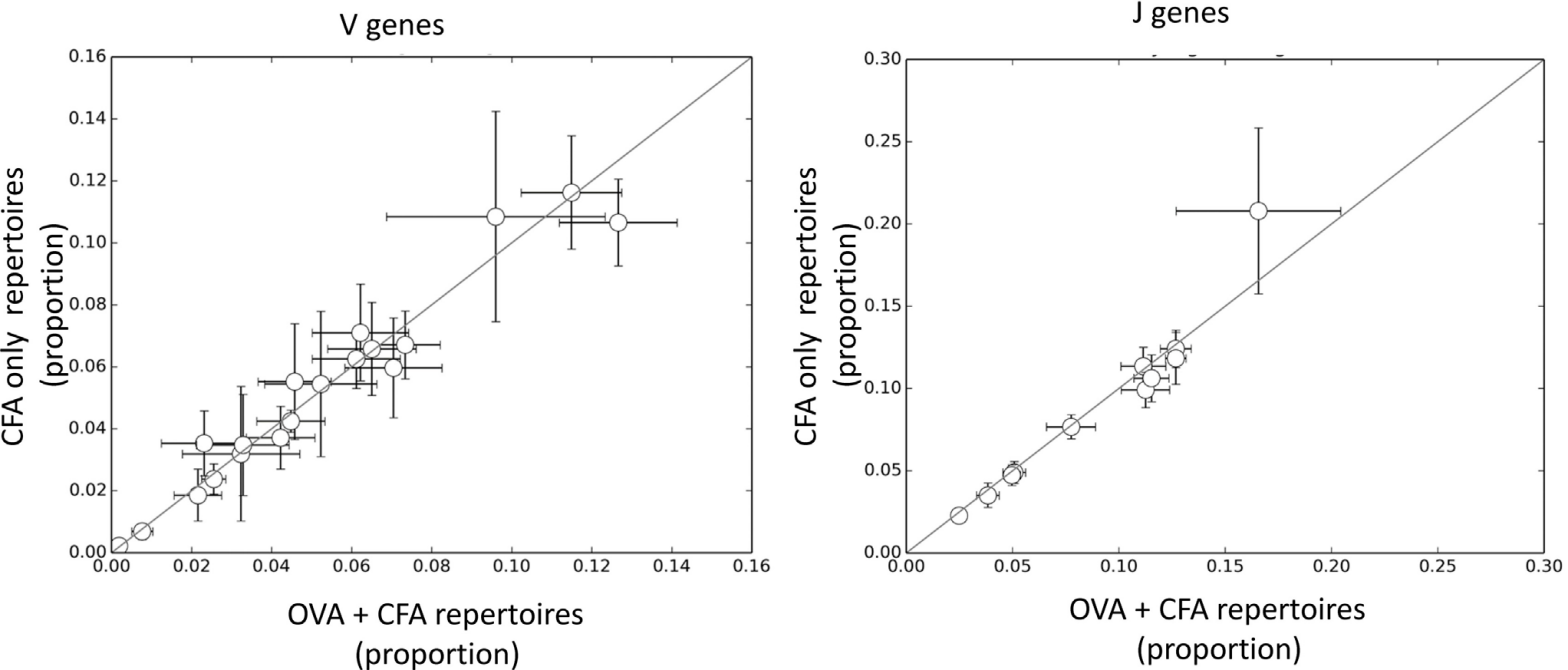
**The frequency of V and J genes in complete Freund’s adjuvant (CFA) versus ovalbumin (OVA) + CFA repertoires**. Each dot shows the frequency of a specific V or J gene in CFA + OVA (*x* axis) and CFA only (*y* axis) repertoires. The error bars show mean ± SD calculated from 50 samples of 10,000 TCRs from each repertoire.

The results presented above suggest that the response to OVA is made up of CDR3βs which are largely private to individual mice and that there is no core set of shared CDR3 sequences which can distinguish between CFA + OVA and CFA repertoires. However, the results also suggest that some sequence features may be enriched within the OVA-associated repertoire. We initially examined the frequency of V or J gene usage between the OVA and the CFA + OVA (Figure 4). However, no individual V or J region differed significantly between the two repertoires. We initially examined the frequency of V or J gene usage between the OVA and the CFA + OVA (Figure 4). However, no individual V or J region differed significantly between the two repertoires.

**Figure 5:**
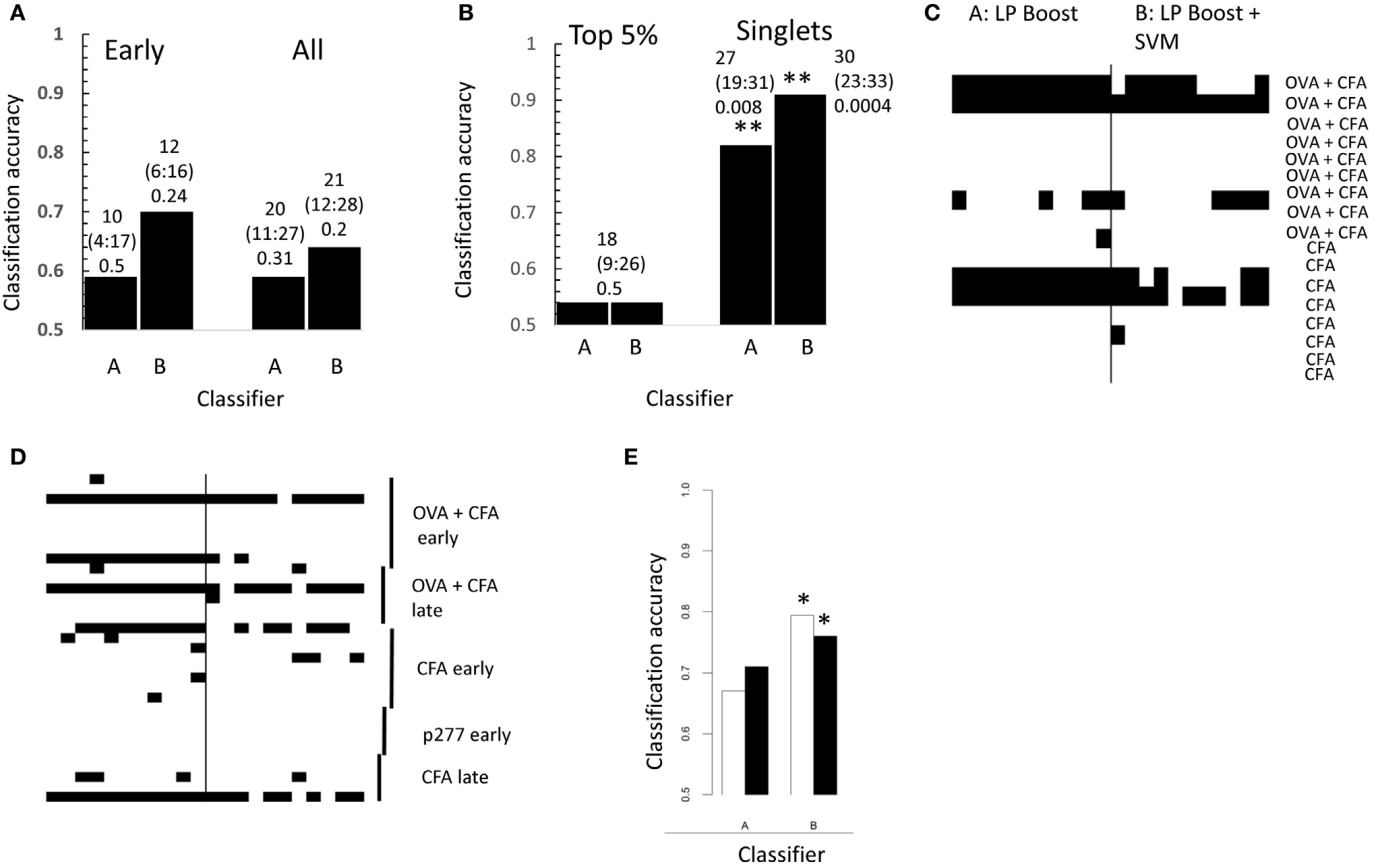
**(A)** Performance of linear programming boosting (LPBoost) with **(B)** or without **(A)** subsequent SVM in the classification of boosting for early (left) and all (right) repertoires. The overall accuracy for each algorithm calculated by majority vote of 99 samples of CDR3s from each repertoire. The results show a trend for correct classification, but are not significantly better than random using Fisher’s exact test. The numbers above each bar show the number of correctly classified repertoires, the 95% confidence range for this estimate, and the Fisher’s exact test *p* value for the result. **(B)** As for panel **(A)** but using only CDR3s within the top 5% percentile of the CDR3 frequency from each repertoire (only one CDR3 sample from each repertoire could be analyzed because of limitation of sample size) or using only clones present once in each repertoire (i.e., singlets). The numbers above each bar show the number of correctly classified repertoires, the 95% confidence range for this estimate, and the Fisher’s exact test *p* value for the result. **p < 0.01 Fisher’s exact test. **(C)** The results of 11 separate subsamples of the early repertoires using singlet CDR3s as in panel **(B)**. Each row of the heatmap represents the repertoire from one immunized mouse, which is omitted from the training set, and then used as test. Each train/test combination was carried out 11 times. Each column of the heatmap therefore represents one replicate train/test cycle. Black indicates incorrect classification. **(D)** As for panel **(C)**, but for all repertoires. **(E)** The filled bars show classification accuracy of LPBoost algorithms on early repertoires using the set of CDR3s with expansion index >6 in any early immunized mouse (cf., Figure 2E). The empty bars show the average classification accuracy obtained from 100 sets (same size) of CDR3s drawn randomly from the combined repertories of all immunized mice. *p < 0.05 Fisher’s exact test.

In order to determine whether the frequencies of triplet amino acid motifs could be used to classify between CFA + OVA and CFA only immunized repertoires, we explored the LPBoost algorithm (LPBoost, see Materials and Methods for details) which produces solutions which are sparse in both samples and features (20). As input for LPBoost we used the frequencies of individual amino acid triplets, as in our previous publication (16). We also combined LPBoost with SVM, by using the features selected by the LPBoost algorithm as input for an SVM. We emphasize that these samples were selected from the complete unselected CDR3 repertoire from each mouse, and not just those CDR3s which were enriched in OVA + CFA repertoires. The performance of these different algorithms using the “early” repertoires (days 5–10) or all the repertoires [to include both later time points, and a different antigen (p277)] is shown in Figure 5A. We obtained modest classification accuracies of between 59 and 71%. With the small sample sizes available these differences are not significantly better than random using Fisher’s exact test. The algorithms gave efficiencies of 52 ± 13% on average if the sample labels were randomized before analysis.

We next explored whether the information required for classification was predominantly found in the more frequent CDR3s, or was distributed among the whole repertoire. The classification performance using only those CDR3 sequences found at high frequency (top 5%, Figure 5B) was consistently poorer than that obtained the whole repertoire (cf., Figure 5A). Unexpectedly, however, classification using clones only appearing once (Figure 5B) gave excellent classification efficiencies using either LPBoost alone or LPBoost plus SVM (*p* < 0.01, Fisher’s exact test; randomized gave 57 ± 11%). The classification was quite stable to repeatedly sampling the data, and some repertories were consistently poorly classified (Figures 5C,D). Overall, information for classification appeared to be distributed across many rare CDR3s suggesting that information reflecting immunization status seems to be quite widely distributed across the CDR3 repertoire.

We also explored whether the more restricted subset of CDR3s with a high expansion index in early immunized repertoires which we identified above (Figure 2E; Figure S2 in Supplementary Material) could provide string triplet features which would improve the classification of the repertoires. The performance of each LPBoost algorithm using either this preselected set of CDR3s or 10 random sets (of same size) of CDR3s is shown in Figure 5E. Using the preselected CDR3s with a high expansion index gave improved classification for 3/4 algorithms, although combining LPBoost with SVM on untransformed string features showed good classification efficiency even on small random sets of CDR3s.

### The Properties of Amino Acid Motifs Selected by LPBoost

**Figure 6:**
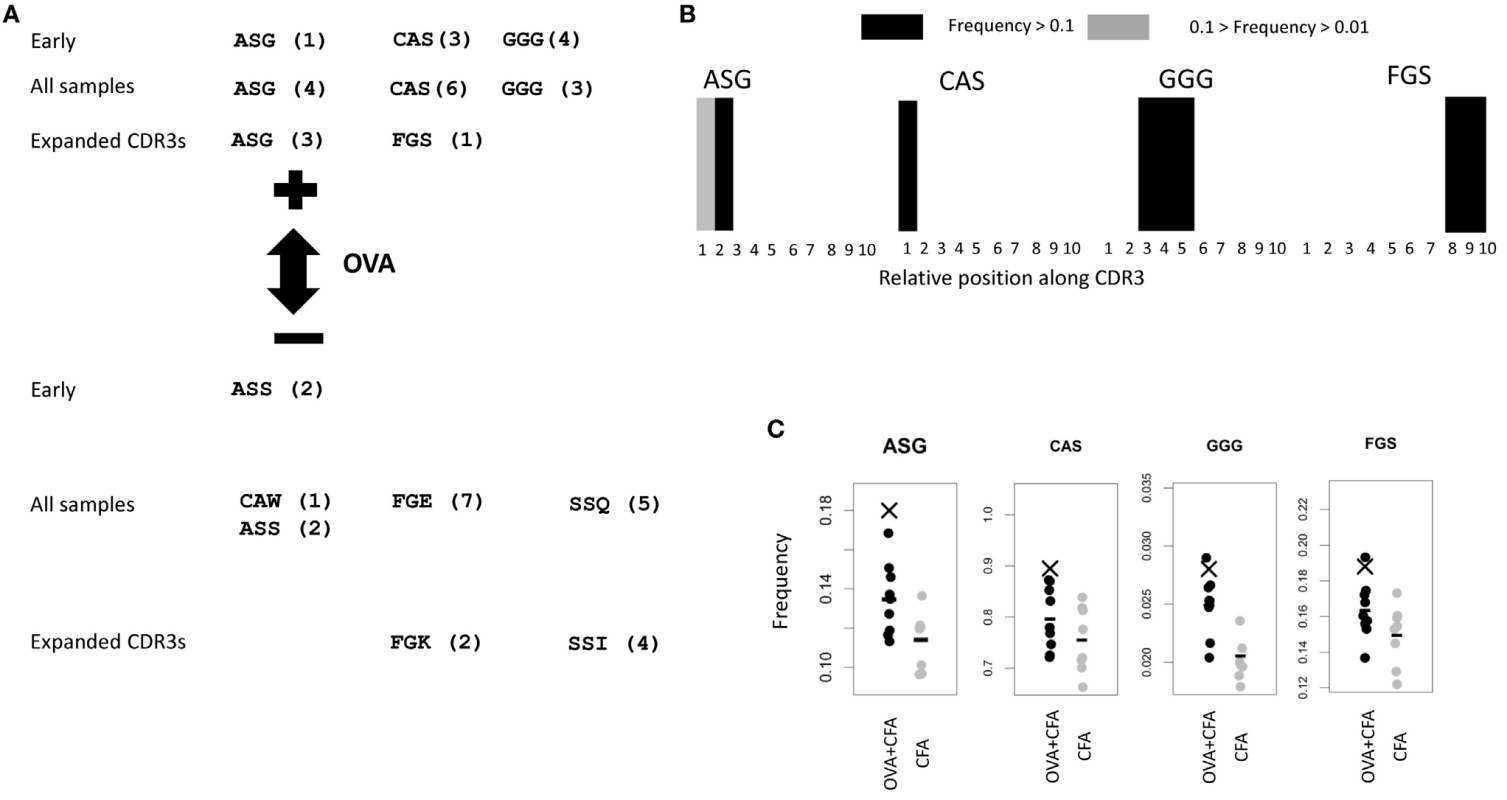
**(A)** Common features selected by linear programming boosting (LPBoost) algorithm (with SVM) using singlet CDR3s (those which appear once only) from repertoires from early repertoires only, all repertoires, and expanded CDR3s as defined in Figure 2D. Only those features selected in a proportion of examples above a threshold are shown. The thresholds are shown in Figure S3 in Supplementary Material. The numbers in brackets show the ranking of the triplet in the plots shown in Figure S3 in Supplementary Material. **(B)** The LPBoost algorithm selects some features with positive coefficients in the equation defining the classifying hyperplane. A higher frequency of these features in a repertoire favors a prediction of an ovalbumin (OVA) + complete Freund’s adjuvant (CFA) classification for that repertoire. By contrast, the LPBoost algorithm selects some features with negative coefficients in the equation defining the classifying hyperplane. A higher frequency of these features in a repertoire favors a prediction of CFA only classification for that repertoire (i.e., it predicts the absence of OVA from the immunization). The panel shows the position of the OVA + CFA predictive triplets along the CDR3. Each CDR3 within a random sample of 10^6^ is divided into 10 equal size units of length (in units of number of amino acids). Individual triplets are assigned a position determined by the relative position of the starting amino acid. The figures show the frequency with which each triplet is found at each position. Black: frequency ≥ 10%; gray: 10% > frequency ≥ 1%; and white: frequency < 1%. **(C)** The frequency (count per 10^4^ CDR3s) of the CFA + OVA-predictive triplets in total early immunized OVA (black) or CFA (gray) repertoires. Bars show average of each population. X shows the frequency of the triplet in the subset of CDR3s which are found at high frequency in OVA repertoires which are illustrated in Figure 3A.

We examined further the properties of those triplets that were selected by the LPBoost algorithm using CDR3s which were present only once, since these gave the best classification efficiencies. The amino acid sequences of these triplets are shown in Figure 6A, together with those triplets selected from the OVA-associated CDR3s. Since slightly different sets of triplets were selected by the algorithm from each different random CDR3 sample of training sequences, and also for each different mouse “left-out,” only those triplets commonly selected are shown (Figure S3 in Supplementary Material). We note that we cannot offer any guarantees that some of these CDR3s were selected by chance and not on the basis of vaccination status. The triplets shown in the top part of the figure have coefficients in LPBoost which positively classify OVA repertoires, while triplets at the bottom negatively classify OVA repertoires (i.e., are associated with non-OVA immunization). The three classifications using CDR3s from early immunized repertoires or all (early and late) immunized repertoires (Figures 5C,D), or using only the subset of 10,175 CDR3s with expansion index >6 (Figure 2E) all identified very similar sets of amino acid triplet features.

We examined where the OVA classifying triplets for the early immunized repertoires were found along the length of the CDR3 (Figure 6B). Strikingly, each triplet showed a highly restricted and non-uniform distribution of expression along the CDR3 [reminiscent of the restricted distributions of amino acids along antibody CDR3s described in Ref. (13)]. The two “OVA-predictive” triplets are found predominantly at the beginning or end of the CDR3s and can be coded for by germline sequences found within some V or J segments (Figure S4 in Supplementary Material). For example, ASG is found in the germ line sequence of TRBV13-2*01. However, 10% of the TCRs which contained the OVA-predictive ASG triplet used Vβ regions other than TRVB13-2*01. In these cases, the ASG sequence has been recreated by deletion and non-template addition. Furthermore, the frequency of TRVB13-2*01 does not differ between OVA + CFA and CFA only repertories (Figure S5 in Supplementary Material). This suggests that the ASG sequence is being selected for independently of V gene.

The frequencies of each of these OVA classifying triplets in each early immunized repertoire for OVA and CFA mice are shown in Figure 6C. The mean frequency of the OVA classifying triplets was higher in the OVA + CFA immunized repertories than in the CFA repertoires. The ASG triplet was also overrepresented in the OVA-associated subset of CDR3s illustrated in Figure 2E. However, the distributions of frequencies in the OVA + CFA and in the CFA alone repertoires were largely overlapping and the frequency of any one triplet alone could not predict immunization status with any degree of accuracy. Each triplet therefore acts only as a “weak learner,” and only a combination of a number of triplets provides good classification.

**Figure 7:**
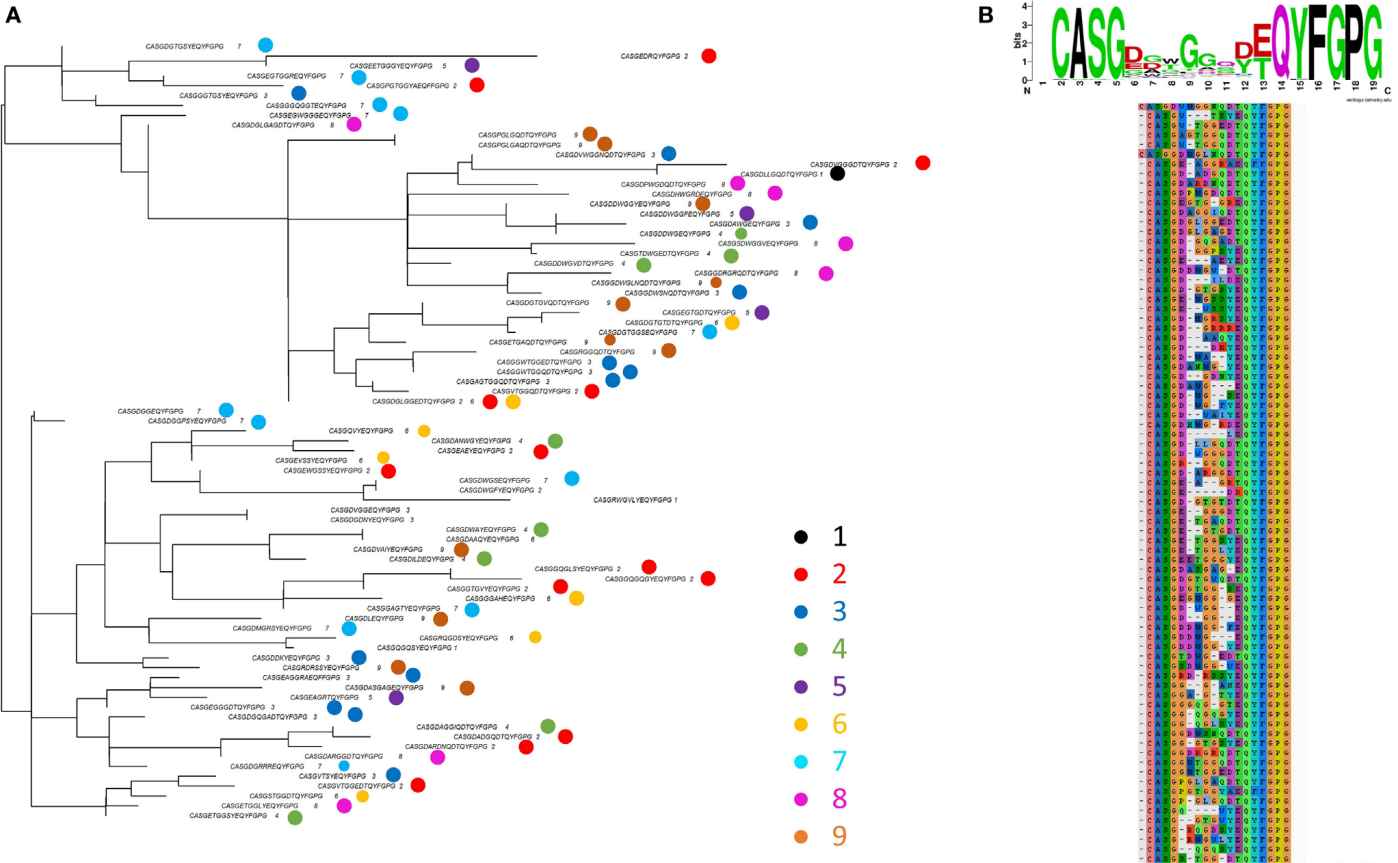
**(A)** One example of a subtree from the whole tree shown in Figure S6 in Supplementary Material. The tip labels show the CDR3s sequence and the individual mouse repertoire(s) from which it was derived (colored according to key). **(B)** Sequence alignment of the CDR3s shown in panel **(A)**.

The ASG triplet was predictive of OVA immunization in analysis of both early and late repertoires and also of the CDR3s with expansion index >6. We therefore extracted the full amino acid sequence for all the “OVA associated” CDR3s shown in Figure 2E which contained ASG. For improved clarity, we show a subtree representing a cluster of CDR3s with more closely related sequence (Figure 7A). A multi sequence alignment of this subset of CDR3s is shown in Figure 7B. The full alignment and a phylogenetic tree representation of these sequences are also available in Figure S6 in Supplementary Material. The set of CDR3s share considerable sequence similarity which extends beyond the defining ASG triplet. As captured by the sequence logo (Figure 7B), the alignment represents a set of highly related CDR3s, many of which share over half their amino acids. The repertoires in which each sequence is found are illustrated by the colored circles in Figure 7A. The sequences are found in one, or occasionally two repertoires, but there is no obvious relationship between the relatedness of the sequence and the repertoire from which they are derived. The ASG motif therefore defines a set of diverse but related sequences of OVA-associated CDR3s. Interestingly, the other OVA selecting triplet FGS is not dominant in this set, suggesting these two triplets are independent predictors of an OVA response.

## Discussion

In the analysis above, we tackle the challenging problem of distinguishing between the T cell repertoires of mice immunized with CFA with and without OVA, by looking only at TCRβ CDR3 sequences. A number of interesting conclusions emerge.

First, our analysis highlights the extraordinary diversity and heterogeneity of the adaptive immune response when considered at a molecular level. In particular, the results show that even when considering beta chains only, the responding set of TCRs in each individual are rarely shared with another individual. This is true even when the individuals are as genetically close as possible, as in the case of the inbred laboratory strain of C57Bl/6 mice considered here, because the diversity arises not from germline diversity but from the vast number of possible outcomes of the somatic stochastic recombination machinery which generates the TCR repertoire in developing T cells. Every individual therefore responds to the same challenge (i.e., OVA in this case) by using T cells expressing differing sets of TCRβs. In fact, each TCRβ will be paired independently with different sets of TCRαs, further expanding the diversity of responding TCRs.

Although at first glance surprising, this conclusion follows on inevitably from a consideration of the quantitative parameters of repertoire generation which have been explored in detail in several recent papers (13, 31, 32). The median probability of finding a particular TCR in mouse TCRβ repertoires is in the order of 10^−9^ [Elhanati, personal communication, cf., Ref. (32) who provide an estimate of 10^−14^ in human TCRβs]. However, each mouse contains only in the order of 50–100 million CD4 T cells. Thus the probability of finding any one particular TCR of “average” generation frequency in a particular mouse is 10^−9^ × 10^8^ = 0.1. To be certain that every mouse will mount an immune response against a specific epitope of OVA, there must be many different TCRs with the *potential* to recognize OVA. Experimental measurements suggest that in the order of at least 20 and as many as two thousand CDR3β can bind a single MHC peptide epitope (4, 33). The inevitable conclusion seems to be that there must be hundreds or even thousands of different potential TCRβ which could form part of a TCR with OVA specificity. In other words, achieving OVA specificity at the level of TCRs requires a highly degenerate solution (34).

The discussion above has emphasized the diversity and uniqueness of different individual’s immune responses to OVA at the level of CDR3β. Nevertheless, OVA recognition ultimately requires a physical/chemical interaction of a certain minimum energy between the surface of the MHC/peptide complex and the TCR antigen binding site. For OVA recognition in mice of the strain C57BL/6 used here, this probably includes a small number of different OVA epitopes which bind efficiently to I-A^b^ MHC molecules. The energy of this interaction will be determined by the (probably non-linear) combinations of interactions at different points of the surface between amino acids of the TCR and amino acids of the peptide in the MHC binding groove (35). It seems likely that achieving a sufficient binding energy for any one particular MHC/peptide target will therefore impose some (perhaps weak) constraints on the structure of binding TCRs, which may be reflected in local sequence features within the CDR3s. Indeed, we observed that the OVA expanded CDR3βs described here are more similar to each other than to same sized sets of CDR3s chosen randomly from the combined repertoires of all the immunized mice. How one might reconcile diversity and degeneracy with specificity is illustrated in the second part of our study, where we provide evidence that particular sets of short amino acid motifs (e.g., contiguous triplets) may encode such binding constraints. We examine this possibility by exploring whether the relative enrichment or depletion of such features, when taken in combination, contains sufficient information to allow classification of the immune status of the repertoire as a whole. Although the methodology we use has been developed primarily in the machine learning framework arising from text or image classification, we are specifically motivated by the emerging consensus that such short local protein sequence motifs may define conserved protein/protein interactions in a much wider context (36, 37).

Our previous attempts to classify immune repertoires using non-zero weighted combinations of all possible triplet frequencies by SVM were successful in distinguishing between unimmunized repertories and CFA immunized repertories. We extend this approach to include an automatic feature selection step, which focuses the classifier on a much more restricted set of features by introducing a 1Norm, in place of a 2Norm term into the optimization algorithm. The optimization can be solved efficiently using a column generation algorithm so that the global optimum solution could be obtained although only a limited number of features need be considered at each step of the algorithm (20). We adapted this approach to the TCR classification problem, and typically obtained optimal solutions which identified some 20–40 non-zero features. We note that the classification results we obtained may be subject to upward bias due to the lack of out-of-sample testing. As such, the classification accuracies should be considered provisional, until validated on a new and independent experiment. Repeat experiments which use improved sequencing protocols, with molecular barcoding and more sophisticated error correction may also lead to improved algorithm performance.

A striking finding was that most amino acid triplets occupy a rather specific location along the CDR3. This presumably reflects the germ line sequences of the V, J, and D region, but may also reflect structural constraints in producing a functional CDR3 loop. A similar observation has been reported for B cells (31). A number of triplets selected by LPBoost were actually formed by the end of the V and J regions (e.g., AGS, CAS, CAW, etc.). The amino acids toward the ends of the CDR3 may therefore play an important role in interacting with the MHC/peptide complex and determining antigen specificity. Alternatively, this association may reflect a hidden statistical association between these sequences and other rarer sequences found within the central area of the CDR3.

One might argue that analysis of the antigen-specific TCR repertoire should start with a simpler problem, for example using a simpler antigen (e.g., a single peptide) in combination with a simpler adjuvant (e.g., LPS or dsDNA) which do not themselves stimulate a significant T cell immune response. The responding T cells could also be purified, for example using MHC multimer technology. Indeed such alternative approaches are extremely valuable and are being pursued both in our own and several other laboratories (33). However, the model we examine is closer to the long term goal of analyzing human TCR repertoires, where immune responses to complex antigens (e.g., viruses or bacteria, which contain a combination of targets of innate and adaptive immunity) must be distinguished against a background of a lifelong exposure to a variety of other immunological stimuli. Furthermore, a global analysis of repertoire does not restrict analysis to T cells binding an individual MHC/peptide epitope, but allows for the possibility that the repertoire will respond more broadly to exposure to specific antigen by readjusting the frequency of both antigen-specific and antigen-non-specific T cells. This allows for such emerging properties as homeostasis, clonal cooperativity, feedback, clonal competition, and cross-reactivity to play a role in shaping the observed TCR repertoire.

In conclusion, we report the results of the first study which uses HTS to examine the global CD4 T cell receptor repertoire of mice immunized with a defined protein antigen in the context of adjuvant. Our results emphasize the extreme diversity of the adaptive immune response at the level of individual antigen-specific receptors, which results in individuals mounting essentially private immune responses. Nevertheless, we also report that the diversity of the responding TCRs may be limited by the requirement for specific sets of amino acid motifs which may be important in determining specificity at a molecular level. Further study of antigen-responsive TCR repertories may shed more light on the rules which govern the physical/chemical interactions between TCRs and MHC/peptide and may ultimately provide tools with which to exploit the TCR repertoire as a sensitive biomarker of an individual’s immune status.

## Ethics Statement

The study was approved by the Weizmann Institute Animal Experimentation Committee.

## Author Contributions

YS, KB, JH, MC, and ES contributed to the computational analysis and to writing the paper. SR-Z and ES contributed to the immunization experiments and TCR sequencing. BC, JS-T, and NF conceived the study, planned the experiments, and wrote the paper.

## Conflict of Interest Statement

The authors declare that the research was conducted in the absence of any commercial or financial relationships that could be construed as a potential conflict of interest.

## Supplementary Material

Figure S1 **The number of shared CDR3 sequences between pairs of mice of different immunization status (as shown on *x* axis), measured as the Jaccard index, calculated using only the top 10% CDR3s ranked according to frequency in each sample**. Squares = early (days 5–14); triangles = late (day 60); and hexagons = early/late comparisons.

Figure S2 **As for Figure 2E but showing those CDR3s with expansion index >6 in any complete Freund’s adjuvant (CFA) repertoire plotted across all ovalbumin (OVA) + CFA or CFA early immunised repertoires**. Each column of the heat map represents one mouse repertoire. Each row of the heat map represents a distinct CDR3 with expansion index >6 in one or more CFA repertoires. Only those positions with an expansion index of >6 in that mouse are colored non-white.

Figure S3 **The proportion of times each feature is selected by linear programming boosting + SVM, in descending ranked order, across all mice and all replicate subsamples shown in Figure 5**. Those features selected >80% of the time (dotted lines) are illustrated in Figure 6.

Figure S4 **Amino acid sequences of the ends of the mouse V and J genes encoding the ends of the CDR3s**. Motifs selected by linear programming boosting as predicting ovalbumin immunization are shown in red.

Figure S5 **The proportion of TCRβs which use TRBV13-2*01 (which codes for the triplet ASG), TRBJ1-2*01, and TRBJ2-3*01 (which code for the triplet FGS) in repertoires from mice immunized with ovalbumin (OVA) + complete Freund’s adjuvant (CFA) or CFA alone**.

Figure S6 **(A)** Alignment of all CDR3s selectively enhanced in early ovalbumin (OVA) repertoire but not in early complete Freund’s adjuvant (CFA) repertoires, which contain the triplet ASG. **(B)** Phylogenetic tree showing relationships of this set of CDR3s. Circle shows subtree expanded in Figure 7.
