# Supplementary figures and images for: Specificity, Privacy, and Degeneracy in the CD4 T Cell Receptor Repertoire Following Immunization
Source: Front Immunol. 2017 Apr 13;8:430. doi: 10.3389/fimmu.2017.00430 (PMC5390035; doi:10.3389/fimmu.2017.00430)

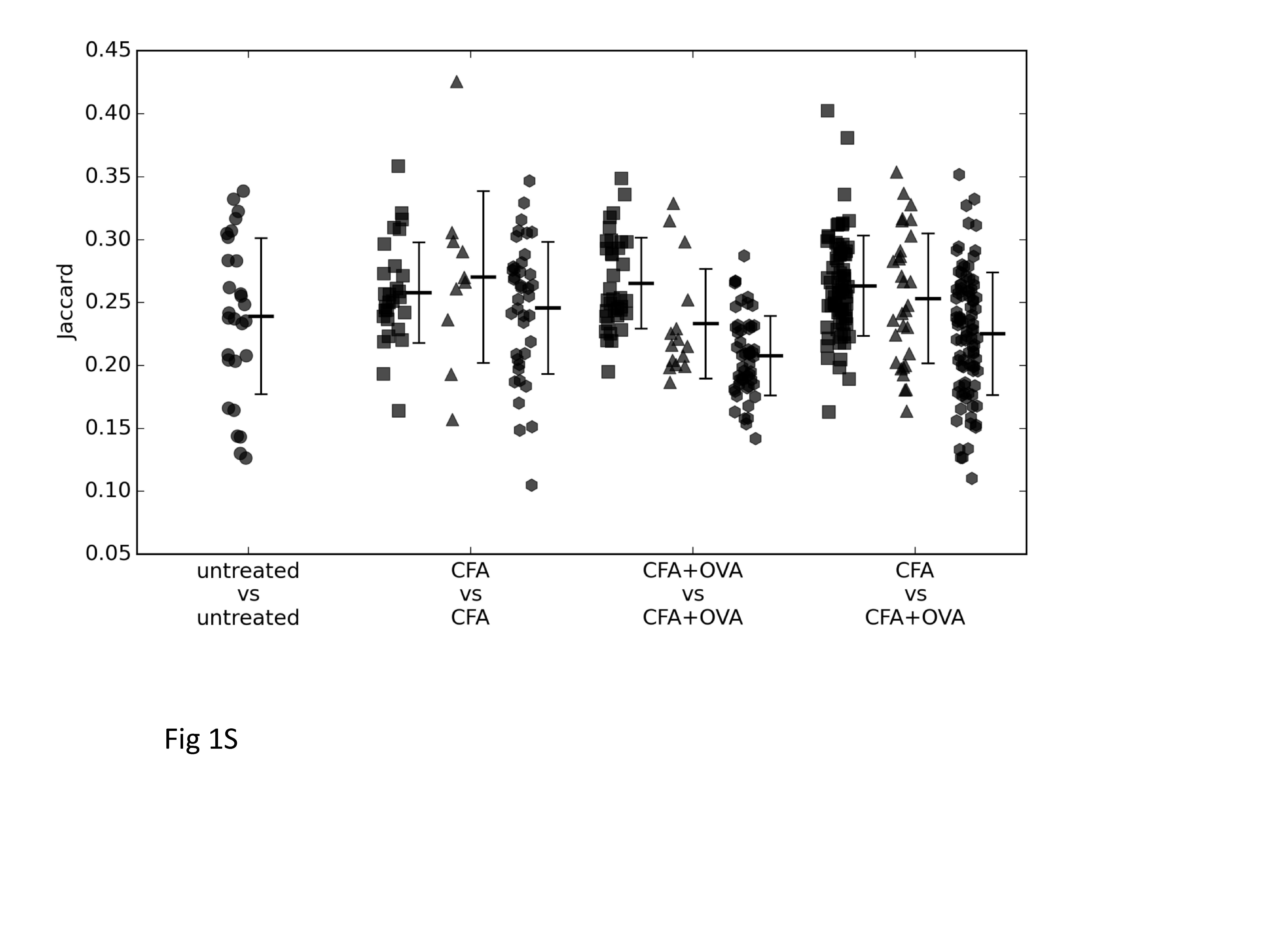

Supplement: Figure S1 — The number of shared CDR3 sequences between pairs of mice of different immunization status (as shown on x axis), measured as the Jaccard index, calculated using only the top 10% CDR3s ranked according to frequency in each sample. Squares = early (days 5–14); triangles = late (day 60); and hexagons = early/late comparisons. [file Image_1.JPEG]

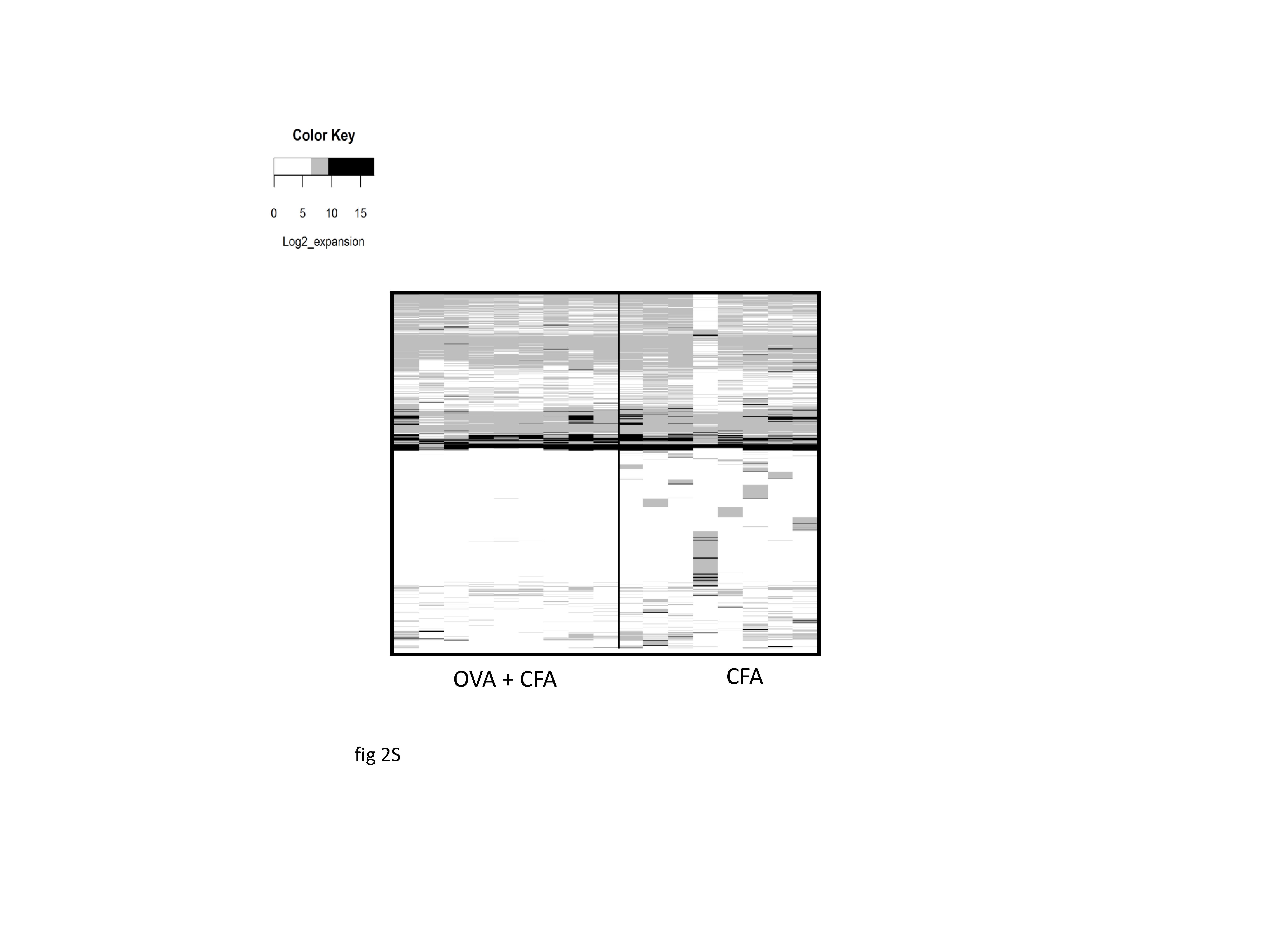

Supplement: Figure S2 — As for Figure 2E but showing those CDR3s with expansion index >6 in any complete Freund’s adjuvant (CFA) repertoire plotted across all ovalbumin (OVA) + CFA or CFA early immunised repertoires. Each column of the heat map represents one mouse repertoire. Each row of the heat map represents a distinct CDR3 with expansion index >6 in one or more CFA repertoires. Only those positions with an expansion index of >6 in that mouse are colored non-white. [file Image_2.JPEG]

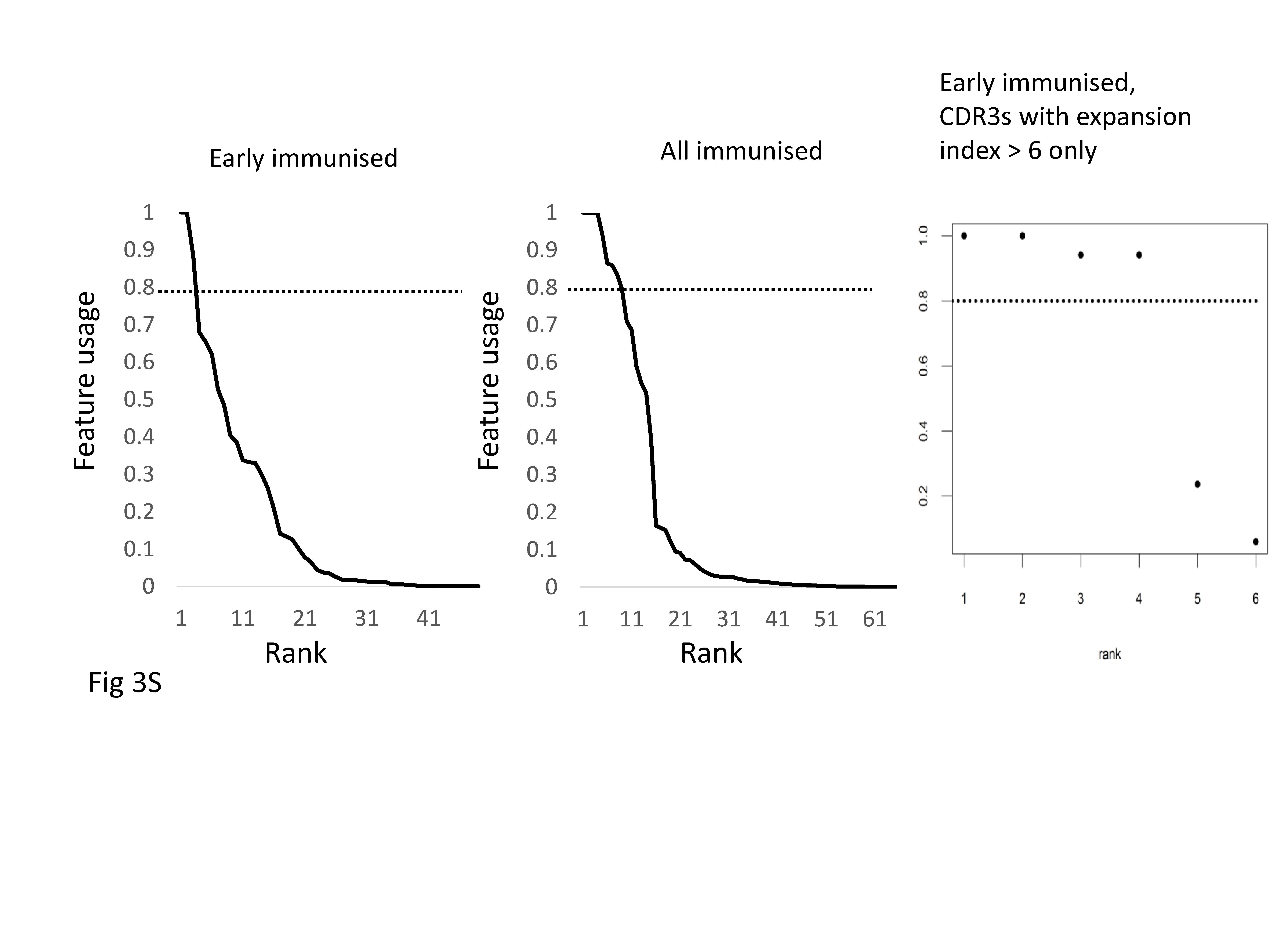

Supplement: Figure S3 — The proportion of times each feature is selected by linear programming boosting + SVM, in descending ranked order, across all mice and all replicate subsamples shown in Figure 5. Those features selected >80% of the time (dotted lines) are illustrated in Figure 6. [file Image_3.JPEG]

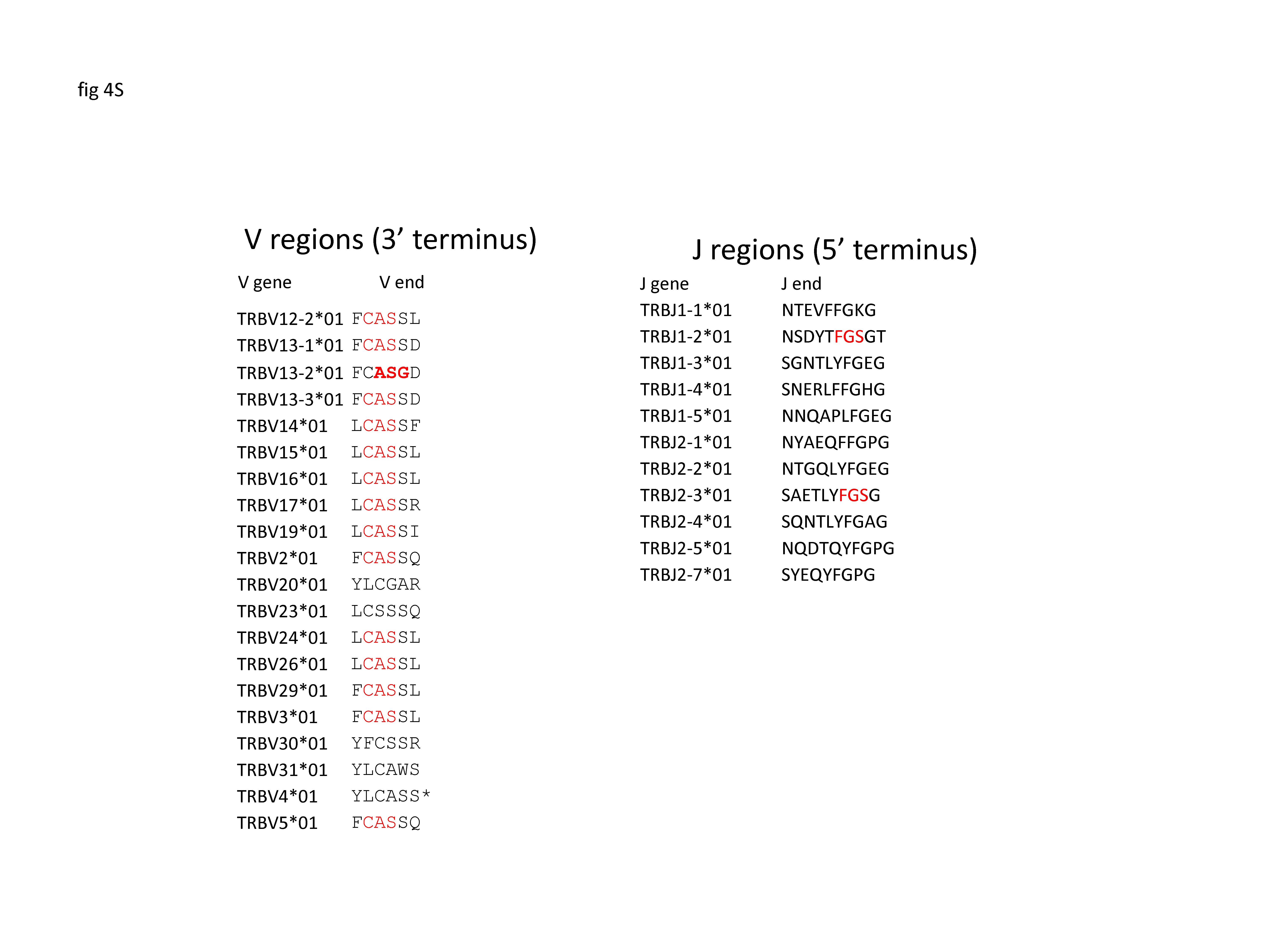

Supplement: Figure S4 — Amino acid sequences of the ends of the mouse V and J genes encoding the ends of the CDR3s. Motifs selected by linear programming boosting as predicting ovalbumin immunization are shown in red. [file Image_4.JPEG]

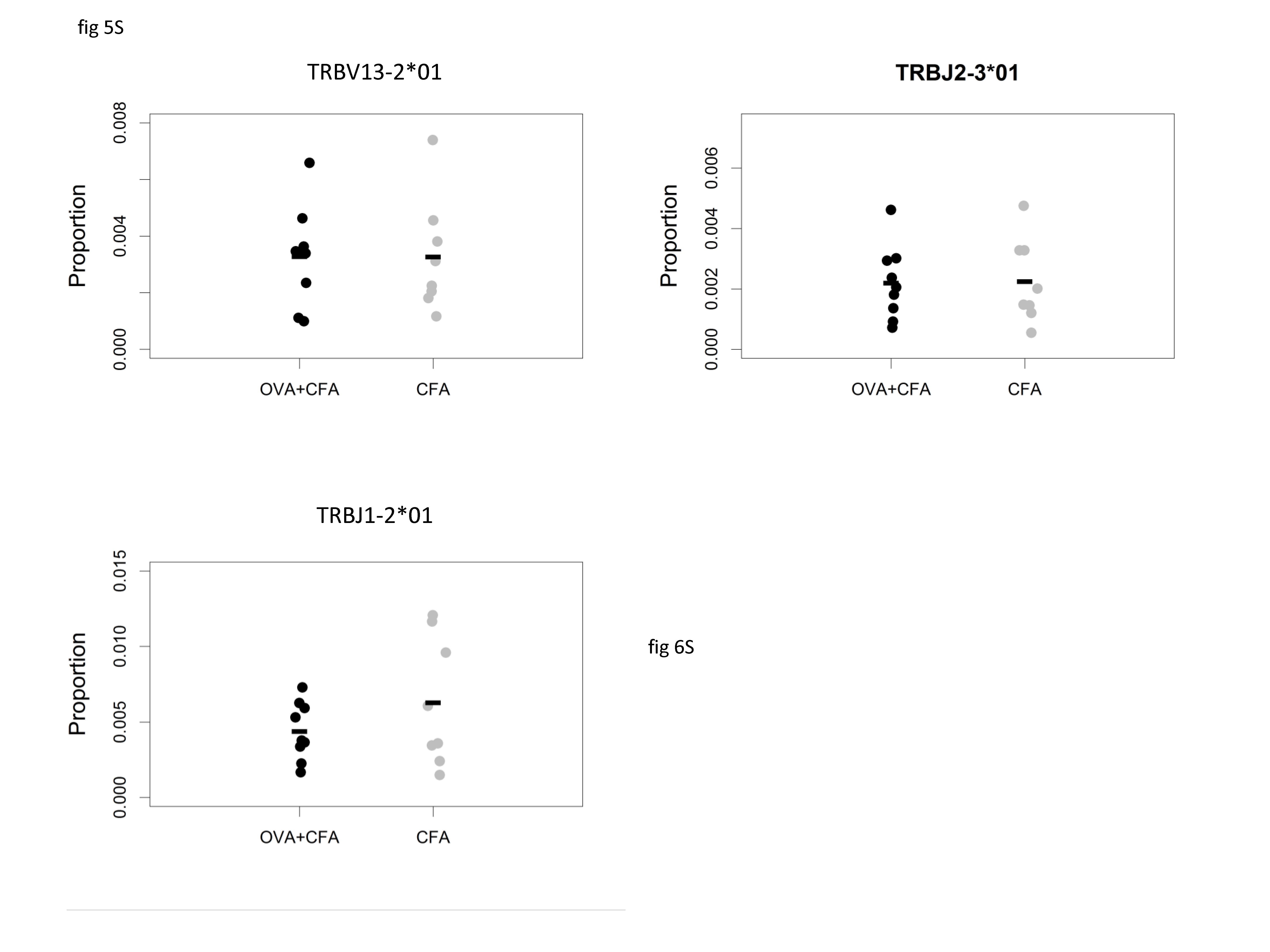

Supplement: Figure S5 — The proportion of TCRβs which use TRBV13-2*01 (which codes for the triplet ASG), TRBJ1-2*01, and TRBJ2-3*01 (which code for the triplet FGS) in repertoires from mice immunized with ovalbumin (OVA) + complete Freund’s adjuvant (CFA) or CFA alone. [file Image_5.JPEG]

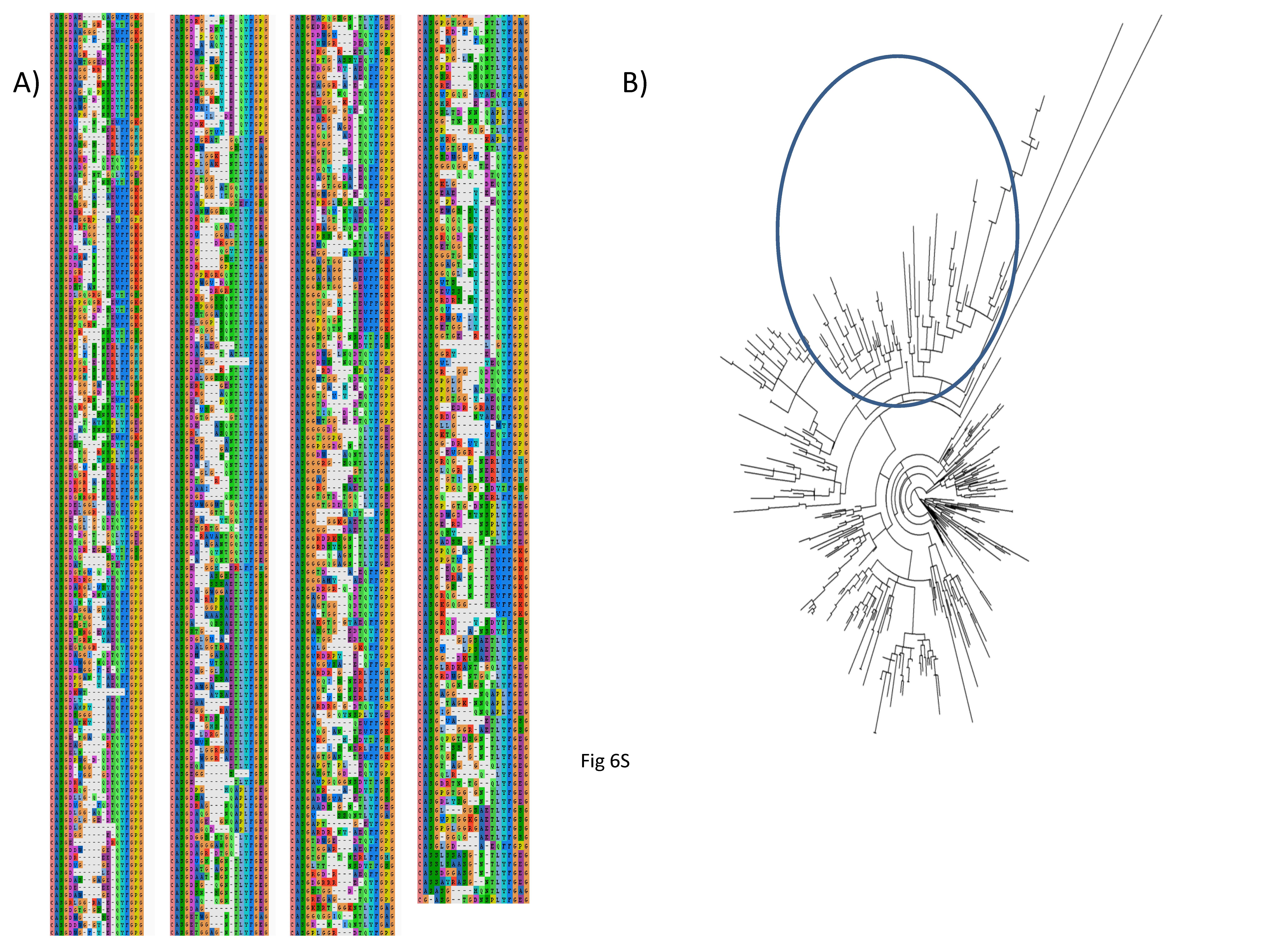

Supplement: Figure S6 — (A) Alignment of all CDR3s selectively enhanced in early ovalbumin (OVA) repertoire but not in early complete Freund’s adjuvant (CFA) repertoires, which contain the triplet ASG. (B) Phylogenetic tree showing relationships of this set of CDR3s. Circle shows subtree expanded in Figure 7. [file Image_6.JPEG]
